# Geometric Modelling for 3D Point Clouds of Elbow Joints in Piping Systems

**DOI:** 10.3390/s20164594

**Published:** 2020-08-16

**Authors:** Ting On Chan, Linyuan Xia, Derek D. Lichti, Yeran Sun, Jun Wang, Tao Jiang, Qianxia Li

**Affiliations:** 1 Guangdong Provincial Key Laboratory of Urbanization and Geo-Simulation, School of Geography and Planning, Sun Yat-sen University, Guangzhou 510000, China; chantingon@mail.sysu.edu.cn (T.O.C.); eesjt@mail.sysu.edu.cn (T.J.); liqianx2@mail2.sysu.edu.cn (Q.L.); 2 Department of Geomatics Engineering, University of Calgary, 2500 University Dr NW, Calgary, AB T2N 1N4, Canada; ddlichti@ucalgary.ca; 3 Department of Geography, College of Science, Swansea University, Swansea SA28PP, UK; yeran.sun@swansea.ac.uk; 4 School of Electrical and Computer Engineering, Nanfang College of Sun Yat-sen University, Guangzhou 510000, China; wangj2@nfu.edu.cn

**Keywords:** elbow joints, geometric model, laser scanning, point cloud registration

## Abstract

Pipe elbow joints exist in almost every piping system supporting many important applications such as clean water supply. However, spatial information of the elbow joints is rarely extracted and analyzed from observations such as point cloud data obtained from laser scanning due to lack of a complete geometric model that can be applied to different types of joints. In this paper, we proposed a novel geometric model and several model adaptions for typical elbow joints including the 90° and 45° types, which facilitates the use of 3D point clouds of the elbow joints collected from laser scanning. The model comprises translational, rotational, and dimensional parameters, which can be used not only for monitoring the joints’ geometry but also other applications such as point cloud registrations. Both simulated and real datasets were used to verify the model, and two applications derived from the proposed model (point cloud registration and mounting bracket detection) were shown. The results of the geometric fitting of the simulated datasets suggest that the model can accurately recover the geometry of the joint with very low translational (0.3 mm) and rotational (0.064°) errors when ±0.02 m random errors were introduced to coordinates of a simulated 90° joint (with diameter equal to 0.2 m). The fitting of the real datasets suggests that the accuracy of the diameter estimate reaches 97.2%. The joint-based registration accuracy reaches sub-decimeter and sub-degree levels for the translational and rotational parameters, respectively.

## 1. Introduction

Pipe elbow joints are essential components in almost all piping systems to support clean water supply, sewage dischargement, petrochemical processes, etc. They are used to connect pipes with different lengths and orientations to form pipe networks. Although geometric modelling of pipe cylindrical bodies was a popular research topic in recent decades (e.g., [1,2,3]), the modelling of elbow joints remains in its infant stage. Pipe system designers often need to know the exact positions and volumes of the joints as they need match the installed joints as closely as possible with specifications and to rate the pipes connected to the joints appropriately [4]. In the field of fluid mechanics, the characteristics of fluid flow (e.g., fluid velocities and pressures) through the elbow joints are of interest for many individuals [5,6,7,8]. Therefore, the geometry of the elbow joints via accurate three-dimensional (3D) modelling is essential for many related designs and studies.

More specifically, the importance of geometric modelling of the elbow joints is that the computation of the Reynolds number (RN) at the joints is required to investigate the flow behaviors [9]. A low RN indicates that the flow is primarily laminar, while a high RN often delineates the presence of turbulence resulting from changes in fluid velocities and directions. The RN depends on the joint diameter which can be affected by the flow pressure [10] and the temperature fluctuation [11]. Therefore, the geometry of the joints should be monitored with various sensors in a way that accurate computation of the RN can be guaranteed. 

Vester et al. [12] measured the flow characteristics to calculate the RN at the exit of several 90° elbow joints of a piping system. They enhanced their measurement method by adding hot wire prob anemometry to capture the complex flow condition at the exit of the joints. Zhang et al. [13] studied the structural vibration and fluid turbulence induced at a 90° elbow joint. Their experiment focused on the impact of guide vanes installed at different positions at the joint, so the geometry of the vanes and joints was accurately known as the preset conditions. Ono et al. [14] investigated the influence of the curvature of the elbow joints on the RN. They concluded that the flow at the joints could be complex as secondary flow can be generated under the interaction between the elbow curvature and separation flow. 

Geometric modelling with parametric equation(s) for 3D pipe components is useful in many other aspects. Zhao et al. [15] proposed a geometric modelling method for underground ferromagnetic pipelines. The elbow joints are modelled in terms of block elements which are generated from breaking down the pipe/joint into many small 3D circular/sectoral units. For each unit, four corners of the sectoral units are expressed in polar coordinate systems defined at the centroid of the pipes/joints. This method focused on characterizing the locations where the magnetic anomalies are present, so their models cannot be readily used for other datasets collected with other sensors such as laser scanners. Moritani et al. [16] modelled pipes with the conventional cylinder modelling approach but analyzed the cylindrical parameters with a proposed 3D hash table in order to match the central axes of the conjugate pipes for point cloud registration. This approach achieved high registration accuracy but did not consider the use of elbow joints. Rabanni et al. [17] developed an integrated feature detection and point cloud registration approach to handle pipe-rich scenes such as industrial sites. They applied more than five types of geometric models to process point clouds of factories which possess complicated piping systems. Nevertheless, their method did not focus on any elbow joints of the systems. These abovementioned research items revealed that the geometric modelling of elbow joints in piping systems has not been fully investigated, but their modelling can provide additional geometric information in addition to the vertical/horizontal pipes. 

The geometric modelling of objects with a defined structure results in many other applications. For example, Chan et al. [18] developed a geometric model for polygonal lamp pole bodies which can be used for point cloud registration. They developed the models to fit the point clouds of lamp poles to estimate accurate parameters. The parameters were then used to generate two sets of correspondents for registration. Voinea et al. [19] proposed a geometric model of the human spine for medical purposes. Accurate geometric models can be used to estimate the volume of an object without complete measurement of the object surface. For example, Chan et al. [20] proposed a set of geometric models for bird eggs. The models were incorporated into a least-squares algorithm which can estimate the volume of eggs based on a point cloud (incomplete) of half of the egg surface obtained from a single scan. Based on the above discussion, it is known that the geometric model of elbow joints not only supports investigation of fluid flow but can also help facilitate other applications such as point cloud registrations and volume estimations.

In this paper, we developed a novel geometric model along with several adaptions for different types of elbow joints of piping systems. This was motivated by the abundance of the elbow joint measurements and high potential of being able to use the 3D joint measurement accurately and efficiently. The paper is organized as follows: Section 2 focuses on the elbow joint model, its adaptation and the paired least-squares fitting method; Section 3 presents the simulated and real datasets along with the experiment; Section 4 and Section 5 deliver the result analysis and conclusion, respectively.

## 2. Geometric Models and Methods

### 2.1. Geometric Models

**Figure 1:**
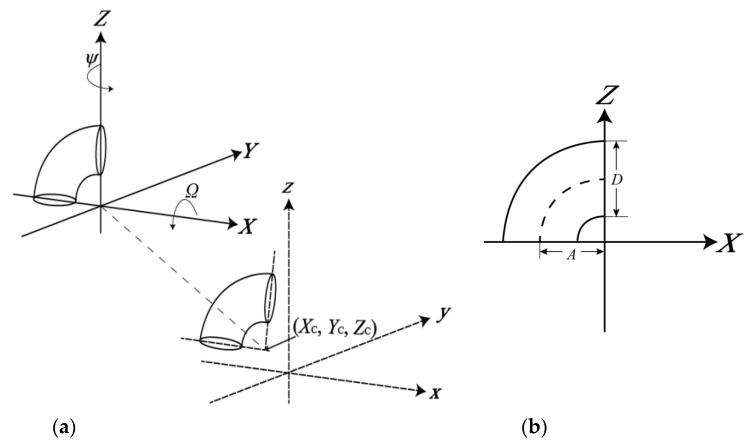
Parameters of the proposed geometric model of the elbow joint (demonstrated with a 90° short-radius joint): (**a**) The translational and rotational parameters; (**b**) The dimensional parameters.

Elbow joints are torus-like structures. The geometry of tori [21] can be used to model the most common types of elbow joints: the 90° short-radius and long-radius joints [4]. When a joint exists in a 3D space or a Cartesian coordinate system, its center (Xc, Yc, Zc) is a translation from the origin of the model space (the coordinate system with solid line axes in Figure 1) and rotation by the vertical/horizontal angles (*Ω*/*Ψ*). As a result, the model of the joints is expressed as follows:

(1) $$f(\vec{x},\vec{l})={\left(Z_{p}-A\right)}^{2}+Y_{p}^{2}-{\left(\frac{D}{2}\right)}^{2}$$

where

(2) $$\left(\begin{array}{c} X_{p}\\ Y_{p}\\ Z_{P} \end{array}\right)=R_{2}(\frac{\pi }{2}-\Phi )\left(\begin{array}{c} X\\ Y\\ Z \end{array}\right)$$

and

(3) Φ=arctan2(Z,X)

and

(4) $$\left(\begin{array}{c} X\\ Y\\ Z \end{array}\right)=R_{3}(\Psi )R_{1}(\Omega )\left[\left(\begin{array}{c} x\\ y\\ z \end{array}\right)-\left(\begin{array}{c} X_{c}\\ Y_{c}\\ Z_{c} \end{array}\right)\right]$$

*A* is the concentric radius of the joint; *D* is the outer diameter of the joint; **R**_1_, **R**_2,_ and **R**_3_ are the rotation matrices about the *X*, *Y,* and *Z*-axes, respectively. Φ is only an intermediate parameter which does not need to be solved during the parameter estimation. Φ is computed for every point lying on the joints for the rotation about the *Y* axis (to lie on the *Y*-*Z* plane) in the model space, so after rotation and the translation of *A* (downward), the point can be fitted to a circle with a diameter equal to *D*.

**Figure 2:**
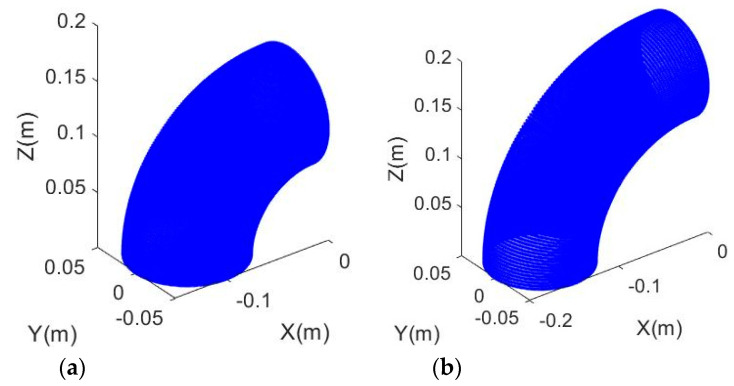
Simulated point clouds: (**a**) The short-radius 90° elbow joint; and (**b**) The long-radius 90° elbow joint.

According to the common standard of the design of the elbow joint [4], the short-radius (namely, 1D in industry) joint is defined as A = D, while the long-radius (namely, A = 1.5D in industry) joint is defined as A = 1.5 × D. A and D can be solved simultaneously if the type of joint is not known. The simulated point clouds for short-radius and long-radius joints with a outer diameter of 10 cm, are shown in Figure 2a,b, respectively.

**Figure 3:**
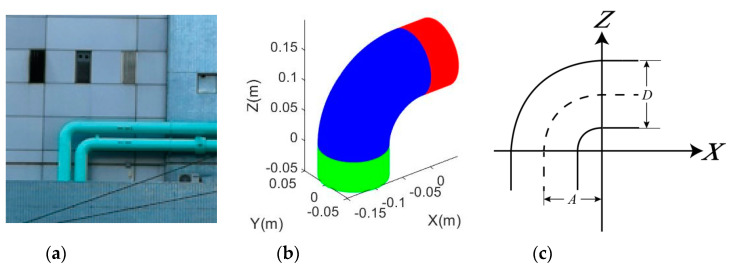
A joint and its connected pipes: (**a**) Examples of joints that are indifferentiable from the connected pipe found in Tsuen Wan, Hong Kong, China. (**b**) Simulated joints with indifferentiable connected cylindrical portions (blue is the bending portion; red and green represent the horizontal and vertical cylindrical portions, respectively); (**c**) dimensional parameters of the adapted model.

Nevertheless, some joints are extended in length close to their connections. More often, in practice, when the joints are surveyed with 3D sensors such as terrestrial laser scanners, certain portions of the connected pipes are inevitably scanned and they are often indifferentiable from the joints (a pipe bend is formed) as illustrated as a real case and a simulated short-radius joint in Figure 3a,b, respectively. These connected cylindrical portions (pipe sections or the match pipes) must be modelled simultaneously with the bending portion with an adapted model (Figure 3c). 

Therefore, we use an index, *p*, namely, the curve index, to allow points lying on the bending portion and the vertical cylindrical portion to rotate about the *Y*-axis when *p* = 1. *p* is expressed as

(5) p=1−sgn(H(X)⋅H(Z))

where *sgn*() and *H*() are the sign and the Heaviside step functions, respectively. Accordingly, Equation (2) is modified as

(6) $$\left(\begin{array}{c} X_{p}\\ Y_{p}\\ Z_{P} \end{array}\right)=R_{2}(p(\frac{\pi }{2}-\Phi ))\left(\begin{array}{c} X\\ Y\\ Z \end{array}\right)$$

The computation of Φ (Equation (3)) can be modified as

(7) $$\Phi =\min\{\theta ,(\frac{\pi }{2}+d)\}$$

**Figure 4:**
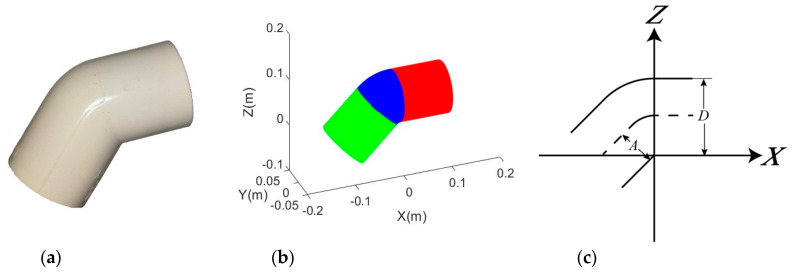
45° elbow joint: (**a**) Real example of a plastic 45° elbow joint; (**b**) Simulated 45° elbow joint (blue is the bending portion; red and green indicate the horizontal and vertical (tilted) cylindrical portions, respectively); (**c**) Dimensional parameters of the adapted model.

θ (0° < θ ≤ 360°) is the angle from the *X*-axis on the *X*-*Y* plane. Equation (7) is able to handle the situation that a point lying on vertical cylindrical portion can be rotated about the *Y*-axis by *π* to intersect the *Y*-*Z* plane so the joint type degree number, *d*, should be set to $\frac{\pi }{2}$ for a 90° elbow. In other words, if *d* is set to $\frac{\pi }{4}$, the model can be used for a 45° elbow as shown in Figure 4.

**Figure 5:**
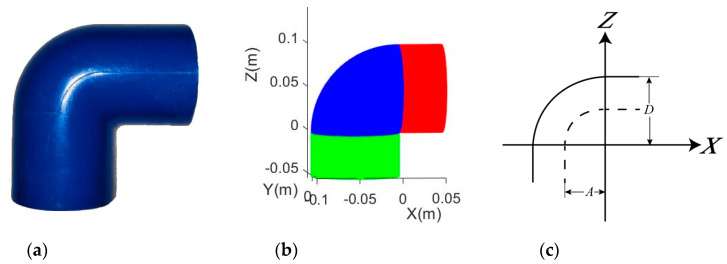
Shape of a 90° elbow joint: (**a**) real example of a plastic sharp 90° elbow joint; (**b**) Simulated sharp 90° elbow joint (blue is the bending portion; red and green represent the horizontal and vertical cylindrical portions, respectively); (**c**) Dimensional parameters of the adapted model.

When *d* = $\frac{\pi }{2}$, the above adaption of the model particularly suits a sharp 90 turn, as shown in Figure 5, which is quite common in piping systems with smaller pipe diameters and fluid pressures. Note that A = 0.5 × D, so we define the nomenclature as A = 0.5*D* herein for consistency even though this is not commonly referred to as this in the industry.

**Figure 6:**
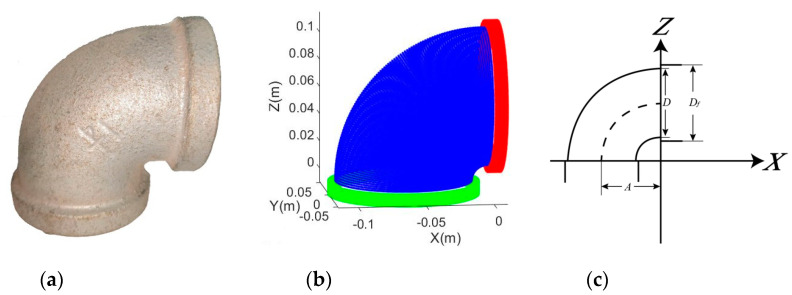
90° elbow joint with flanges: (**a**) Real example of an elbow joint with flanges; (**b**) Simulated elbow joint with flanges (blue is the bending portion; red and green represent the horizontal and vertical flanges, respectively); (**c**) Dimensional parameters of the adapted model.

Finally, for modelling a joint with flanges having a larger outer diameter (*D_f_*), as shown in Figure 6, Equation (1) can be modified to adapt to this additional diameter:

(8) $$f(\vec{x},\vec{l})={\left(Z_{p}-A\right)}^{2}+Y_{p}^{2}-{\left[(1-q)\frac{D}{2}+q\frac{D_{f}}{2}\right]}^{2}$$

Here, we require an index, *q*, namely, the flange index, to allow points lying on the flange to be associated with its larger diameter, $D_{f}$. *q* is expressed as

(9) $$q=H\left(\theta -\left(\frac{\pi }{2}+d\right)\right)+H\left(\frac{\pi }{2}-\theta \right)$$

*q* = 1 for a point lying either on the vertical or horizontal flange. The first term is the vertical flange, while the second term is the horizontal flange.

### 2.2. Least-Squares Fitting

#### 2.2.1. Linearized Adjustment Model

The observations ($\vec{l}={\left(x\;y\;z\right)}^{T}$) obtained by simulation or laser scanning were fitted to the proposed model (Equation (1) or (8)) to estimate the parameters. Since the observations and parameters are not separable in the equation, the Gauss–Helmert adjustment model [22] was employed. The linearized adjustment model is given by

(10) $$A\hat{\delta }+B\hat{v}+w=0$$

where $\hat{\delta }$ is the correction vector for the parameter vector $\vec{x}$ = $\left[\begin{array}{cc} \begin{array}{cc} \begin{array}{cc} X_{c} & Y_{c} \end{array} & \begin{array}{cc} Z_{c} & \Omega \end{array} \end{array} & \begin{array}{cc} \begin{array}{cc} \Psi & A \end{array} & \begin{array}{c} D \end{array} \end{array} \end{array}\right]$; or $\vec{x}$ = $\left[\begin{array}{cc} \begin{array}{cc} \begin{array}{cc} X_{c} & Y_{c} \end{array} & \begin{array}{cc} Z_{c} & \Omega \end{array} \end{array} & \begin{array}{cc} \begin{array}{cc} \Psi & A \end{array} & \begin{array}{cc} D & D_{f} \end{array} \end{array} \end{array}\right]$; **A** is the design matrix of partial derivatives of the proposed model with respect to the parameters; **B** is the design matrix of partial derivatives of the proposed model with respect to observations, $\hat{v}$ is the residual vector; and *w* is the misclosure vector.

#### 2.2.2. Initial Value Estimation

**Figure 7:**
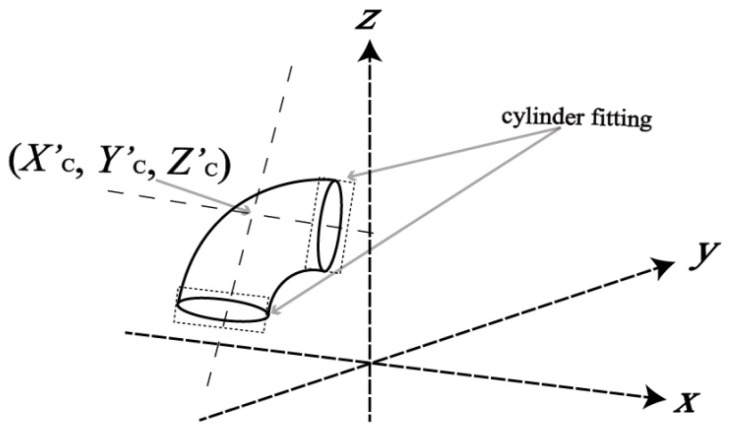
Computation of the joint inner center using two sets of cylinder fittings.

An accurate set of initial values of the model parameters is often needed to guarantee the convergence and the accuracy of the least-squares solution. A straightforward approach based on cylinder fittings is proposed to estimate the initial values of the parameters. Since the joint was detected by first segmenting the connected pipe based on the Principal Component Analysis (PCA) method [23], the approximate position of the joint-pipe connection is known. Then, we extracted a thin slice of points (about 1 cm thick) near connections to input to the cylinder fitting [24] as shown in Figure 7. An inner center ($\begin{array}{ccc} X_{c}^{\prime } & Y_{c}^{\prime } & Z_{c}^{\prime } \end{array}$) was readily computed by estimating the intersection between two central axes of the best fit cylinders. After that, the joint was translated to the inner center, and the golden section search algorithm [25] applied to estimate initial values of *Ω* and *Ψ* by setting the rotations about an axis passing through the inner center and the *X*-*Y* plane [20]. Finally, the initial of A is the distance between the original and the inner center. The initial values of ($\begin{array}{ccc} X_{c}^{} & Y_{c}^{} & Z_{c}^{} \end{array}$) can be computed by translating the inner center along the axis between the inner center and the origin by *A* toward the origin. The initial value of *D* is naturally obtained from the previous cylinder fitting.

### 2.3. Applications

As dicussed in Section 1, the model can be potentially used to facilate monitoring of the geometry of the elbow joints in piping sytems to help invesitagation of the flow of fuilds. However, our focus in this paper is on the geospatial analysis of the model and its related applications such as point cloud registration and mounting bracket detection.

#### 2.3.1. Point Cloud Registration

After the parameters are estimated by using the model, the translational and rotational parameters can be used to transform the joint to its nominal position in the model space. Therefore, two point clouds of the same joint collected at two different stations can be registered to a common coordinate system. Suppose the estimated parameter vectors of the two point clouds of the same joint, Point Clouds 1 and 2, are ${\vec{x}}_{1}$ = $\left[\begin{array}{cc} \begin{array}{cc} \begin{array}{cc} X_{c1} & Y_{c1} \end{array} & \begin{array}{cc} Z_{c1} & \Omega_{1} \end{array} \end{array} & \begin{array}{cc} \begin{array}{cc} \Psi_{1} & A_{1} \end{array} & D_{1} \end{array} \end{array}\right]$ and ${\vec{x}}_{2}$ = $\left[\begin{array}{cc} \begin{array}{cc} \begin{array}{cc} X_{c2} & Y_{c2} \end{array} & \begin{array}{cc} Z_{c2} & \Omega_{2} \end{array} \end{array} & \begin{array}{cc} \begin{array}{cc} \Psi_{2} & A_{2} \end{array} & D_{2} \end{array} \end{array}\right]$, respectively. Point Cloud 2 (x,y,z) can be registered to the coordinate system of Point Cloud 1 by using the following equation:

(11) $$\left(\begin{array}{c} x^{\prime }\\ y^{\prime }\\ z^{\prime } \end{array}\right)={\left[R_{{}_{3}}(\Psi_{1})R_{1}(\Omega_{1})\right]}^{-1}\{R_{3}\left(\Psi_{2}\right)R_{1}(\Omega_{2})\left[\left[\begin{array}{c} x\\ y\\ z \end{array}\right]-\left(\begin{array}{c} X_{c2}\\ Y_{c2}\\ Z_{c2} \end{array}\right)\right]\}+\left(\begin{array}{c} X_{c1}\\ Y_{c1}\\ Z_{c1} \end{array}\right)$$

Overall, the point cloud registration can be performed as long as two point clouds of the same joint obtained from two stations are fitted to the model to estimate the parameters. Point clouds of only one single joint are required for the registration. 

#### 2.3.2. Mounting Bracket Detection

**Figure 8:**
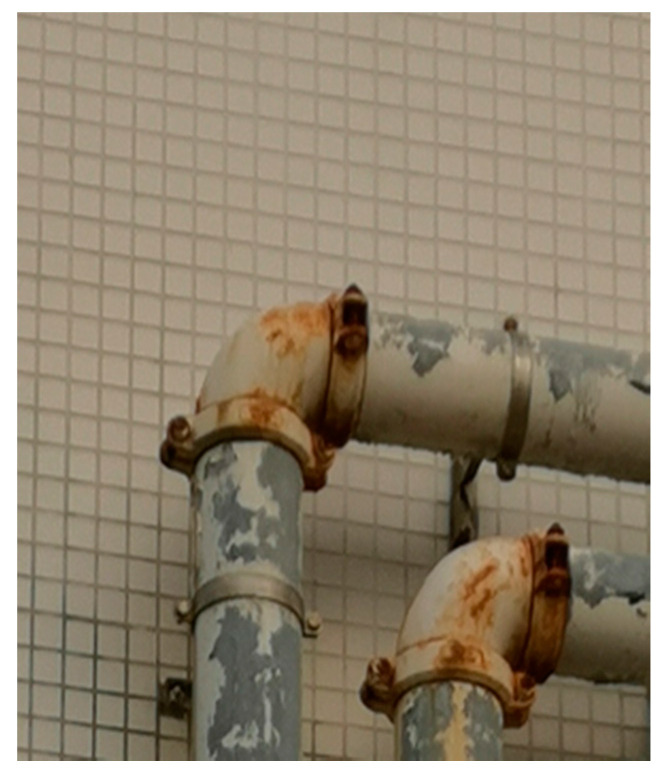
Mounting brackets near two elbow joints found in Yuen Long, Hong Kong, China.

A point which completely lies on the model (the modelled surface) of the joints will possess zero residual under the assumption that the parameters are perfectly estimated. In other words, a point which is farther away from the model will tend to have higher residuals. As a result, any pipe accessories (e.g., a mounting bracket as seen in Figure 8) that adhere to the joint surfaces can be detected by observing the magnitudes of the residuals. A simple way to implement a mounting bracket detection algorithm is by setting a threshold for the residual magnitude. After the points with larger residuals are detected, they need to be grouped together and filtered. Similar to the point cloud processing framework proposed by Wang et al. [26], we employed Density-Based Spatial Clustering of Applications with Noise (DBSCAN) to group the points with higher residuals and then selected the largest segment and the Random Sample Consensus (RANSAC) cylinder fitting to refine the point clouds to segment out the mounting bracket.

## 3. Experiment

### 3.1. Simulated Datasets

Eight elbow joints were simulated as 3D point clouds, and their details are listed in Table 1. Each joint is a full joint with 360° coverage and has the same number of points (47160) and an outer diameter (D) of 0.1 m. Some of the joints were re-simulated with a higher diameter (D = 0.2 m) and with different coverages (from 10° to 360°) to support further analysis.

### 3.2. Real Datasets

**Figure 9:**
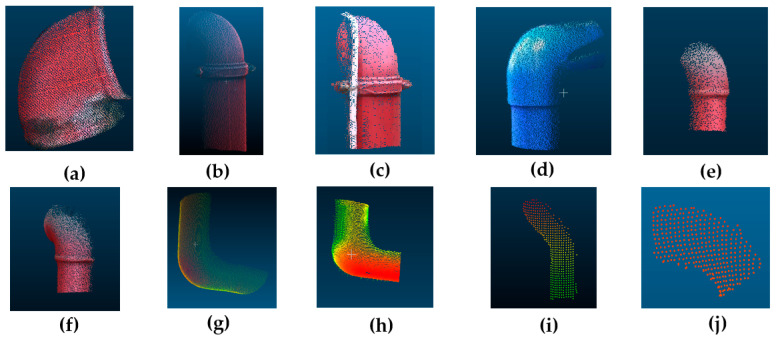
Real datasets collected by using laser scanners: (**a**) Joint A; (**b**) Joint B; (**c**) Joint C; (**d**) Joint D; (**e**) Joint E; (**f**) Joint F; (**g**) Joint G; (**h**) Joint H; (**i**) Joint I; (**j**) Joint J.

**Figure 10:**
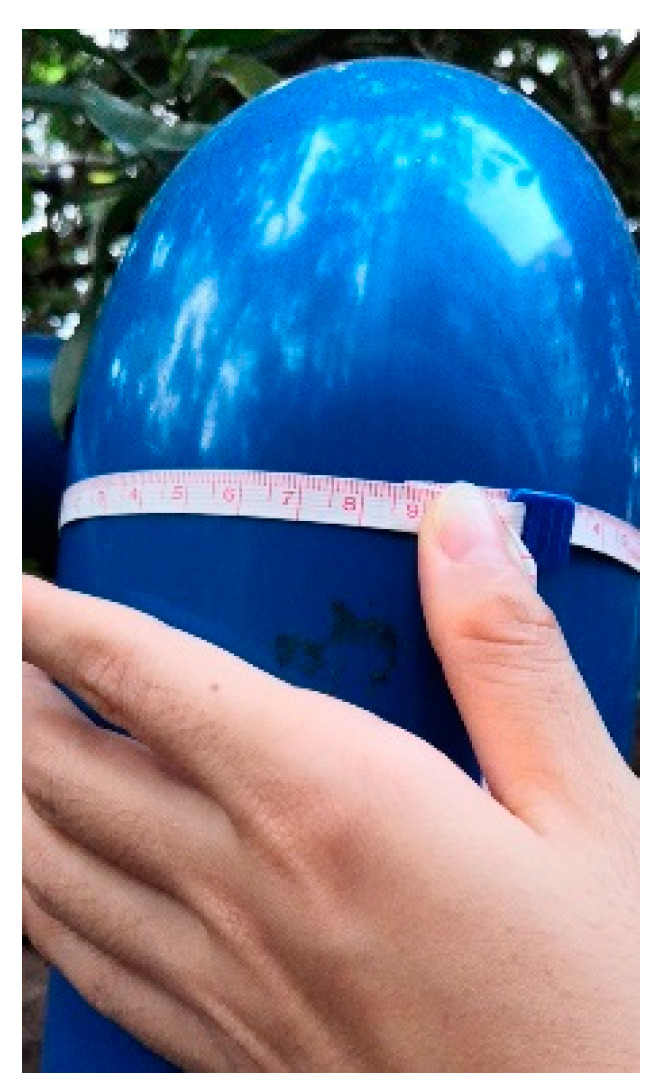
Manual measurement with tape for the circumference (outer diameter) of a joint section.

Ten point clouds of elbow joints were collected with two laser scanners, and the details are shown in Table 2. All the joints were extracted from the entire point clouds based on the PCA method [23] and some manual editing. Joints A-F (Figure 9a–e) were captured by a Trimble SX 10 scanner (a product of the Trimble Inc., Sunnyvale, CA, USA) on the Guangzhou campus of the Sun Yat-sen University, China, in July 2019, while Joints G-J (Figure 9f–h) were captured by a Faro Focus^3D^ scanner (a product of the Faro Technologies Inc., Lake Mary, FL, USA) at the ICT building of the University of Calgary, Calgary, AB, Canada, in May 2014. The diameters of the joints were obtained by tape measurement for reference (Figure 10). 

## 4. Results

### 4.1. Model Analysis with Simulated Datasets

#### 4.1.1. Accuracy Analysis

The root mean square error (RMSE) of the translation parameters (${\hat{\delta }}_{T}$) and the rotational parameters (${\hat{\delta }}_{R}$) estimated from the fittings of the simulated datasets are tabulated in Table 3. The RMSE is computed using the estimated and the true (pre-set) parameter values. It can be seen that the fitting of the models can recover the parameters with 100% accuracy when there is no noise added to the point clouds. As higher noise levels (from ±1 mm to ±3 mm) are introduced to the point clouds, the RMSEs increase as expected. ${\hat{\delta }}_{T}$ stays at a sub-millimeter level except for Joints c and g which are both the 0.5D types. This is because there is an intersection between the bending and the two cylindrical portions of the joint for the 0.5D type. The noise is caused by the points from the three portions mixed at the intersection, and therefore degrades the accuracy.

Similarly, ${\hat{\delta }}_{R}$ stays at a sub-degree level except for Joints (c) and (g). Table 4 shows the RMSEs re-estimated by using the same set of simulated joints with doubled A/D (fixed D = 0.2 m) and a raised noise level (from ±3 mm to ±6 mm). It can be seen that the RMSEs of Joints (c) and (g) are significantly reduced at a noise level of ±3 mm. However, the fitting failed to estimate the parameters (the least-squares algorithm did not converge) when the noise levels are further increased as seen in Table 4. From the tables, it is known that larger joints (larger D) tend to possess higher accuracy of the estimated parameters. Another conclusion is that 90° joints can deliver better fitting results compared to the 45° joints thanks to the fact that more observations can be obtained in the bending parts for the 90° joints.

The RMSE of the dimensional parameters (${\hat{\delta }}_{AD}$) for the joints (D = 0.2 m) is shown in Table 5. Compared to Table 4, the accuracy of the dimensional parameters is even better than the translational parameters, especially when the ±6 cm noise level is introduced to the point clouds. This indicates that the model can estimate the dimensional parameters more accurately than the translational parameters under higher noise levels. So far, Joints b and f have the lowest errors for the respective 90° and 45° joints at the ±6 mm noise level. To investigate the noise level the model can bear, noises from ±20 mm to ±80 mm were introduced to the joints (D = 0.2 m). Surprisingly, as seen in Table 6, the model can still deliver quite accurate translational and rotational parameters with errors of only 1.1 mm and 1.072°, respectively, even though ±80 mm noise was introduced to a joint with a of diameter of 200 mm 

**Figure 11:**
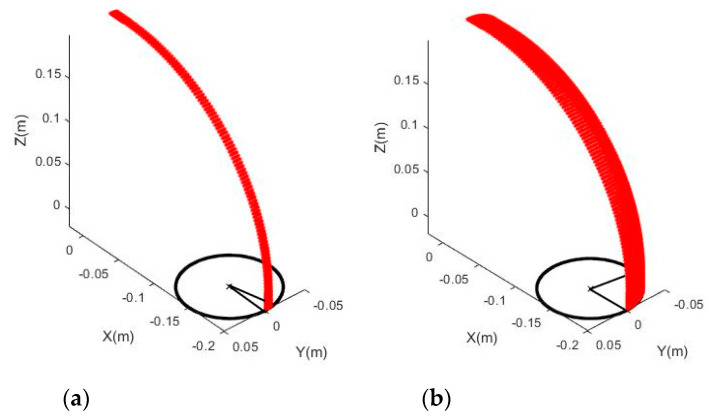

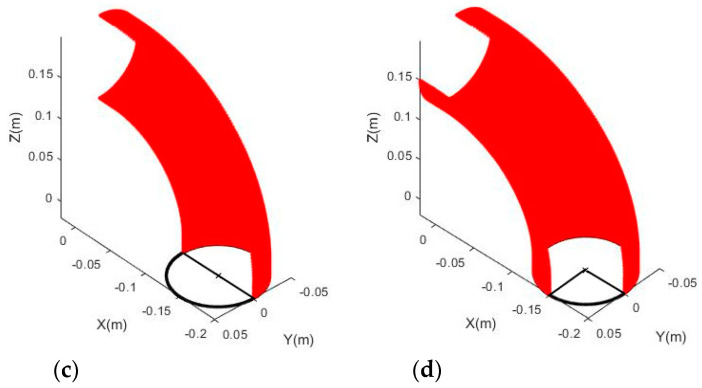
Simulated joint (Joint (a)) with: (**a**) 10°, (**b**) 90°, (**c**) 180°, and (**d**) 270° coverage.

In practice, one scan can only capture about half of a joint, so mostly only about half of the full point clouds of a joint can be fitted to the model unless the point clouds are registered before with two or more scans. Therefore, it is important to investigate the impact of the coverage of the point cloud of a joint on the model accuracy. Joint (a) (90°, 1D, D = 0.1 m) was re-simulated from 10° to 360° at an interval of 10° (simulated joints with 10°, 90°, 180°, and 270° coverage are shown in Figure 11), and different noise levels were introduced to the simulated point clouds.

**Figure 12:**
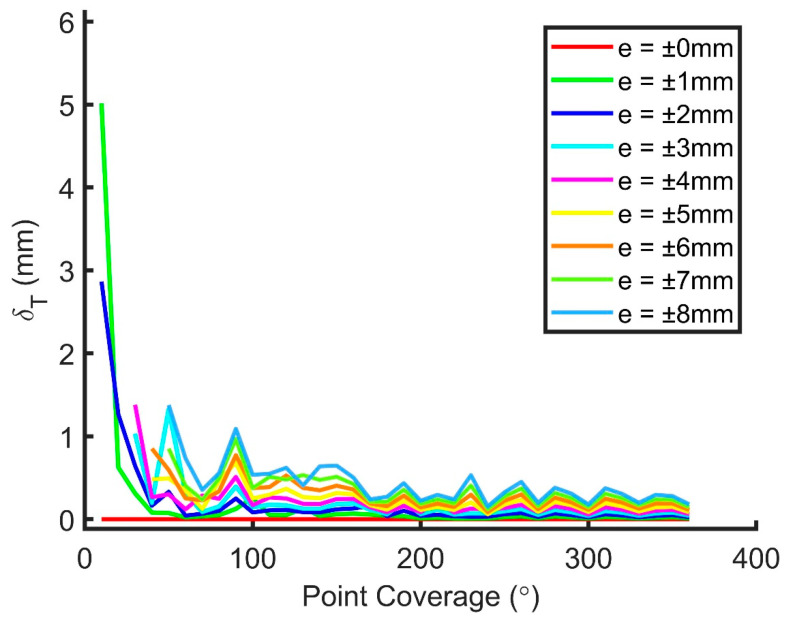
RMSE of the translational parameters for Joint (a) from 10° to 360° coverage and from noise level 0 mm to ±8 mm.

**Figure 13:**
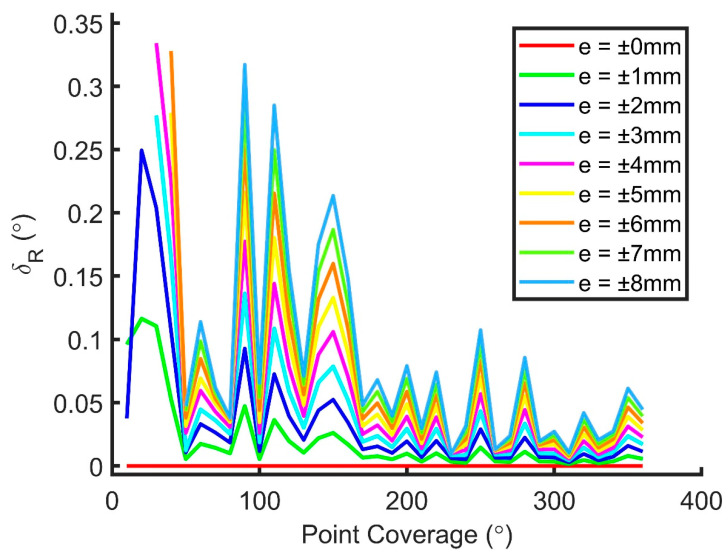
RMSE of the rotational parameters for Joint (a) from 10° to 360° coverage and from noise level 0 mm to ±8 mm.

**Figure 14:**
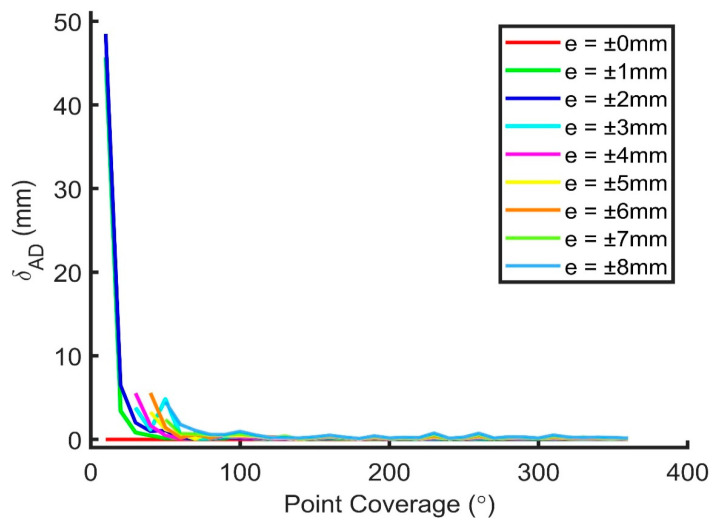
RMSE of the dimensional parameters for Joint (a) from 10° to 360° coverage and from noise level 0 mm to ±8 mm.

The RMSE of the translational, rotational, and dimensional parameters for Joint (a) from 10° to 360° coverage and from noise level 0 mm to ±8 mm is shown in Figure 12, Figure 13 and Figure 14, respectively. It can be seen that all three RMSE values decrease to a low level at 180° or higher coverage (less than approximately 0.5 mm, 0.1°, and 1 mm for the translational, rotational, and dimensional parameters, respectively) so that observations obtained from a single scan (about 180° coverage) can be fitted well to the model theoretically. The rotational parameters are relatively more prone to the change of the coverage and random noises. From the figures, it is known that the increments of random noises from 0 to ±8 mm do not lead to large increases in the RMSEs. Results of scanner calibrations published recently [27,28,29,30,31] indicated that the point cloud errors are normally less than 8 mm within a scan range of 10 m. As a result, it is deduced that the proposed model can work well with half of the joint scanned from a single scan. 

#### 4.1.2. Precision Analysis

The mean estimates and the precision of Joints (a) and (d) with different coverages are tabulated in Table 7. The precision increases as the coverage increases. This is as expected because the degree of freedom increases as the coverage increases. 

At low coverage (90°) of the simulated points, the precision of the dimensional parameters is lower than that of the translation parameters, but this situation reverses as the coverage increases. When the coverage is only 90° (Figure 11b), the precision of D will be quite low as observations are only from one quarter of a circle, so this significantly degrades the dimensional parameters. On the other hand, it can be seen from Table 7 that the precision of the 45° joint is lower than that of the 90° joint. This is due to the fact that the bending portion of the 45° joint is shorter than that of the 90° joint. The shorter bending portion contributes less geometrical information to the estimation so a lower precision is obtained.

### 4.2. Model Analysis with Real Datasets

#### 4.2.1. Estimated Parameters and Precision

The mean estimates and the precision of the real dataset are shown in Table 8. The precision is high and has the same order of magnitude as the precision of the simulated datasets (180° coverage) shown in Table 7. All fitting converged within 10 iterations. The mean estimated joint diameter is 122.55 mm and its accuracy reaches 97.2% when it is compared to manual measurements. This suggests that the proposed model accurately fitted the real data obtained from the scanners. 

#### 4.2.2. Point Cloud Registration

**Figure 15:**
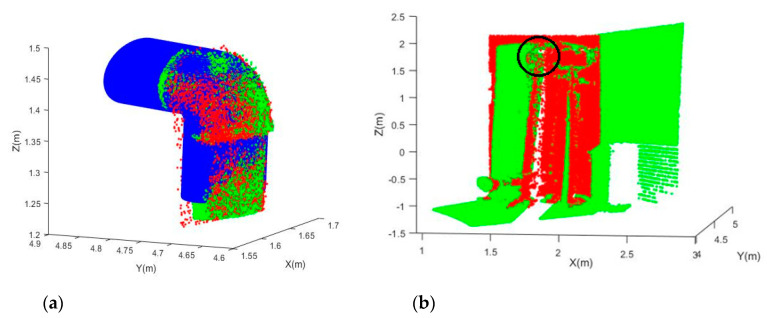
Registered point clouds: (**a**) The registered Joints E-F (red for Joint E, green for Joint F, and blue for the model); (**b**) The entire registered point cloud (the black circle shows the joint position).

**Figure 16:**
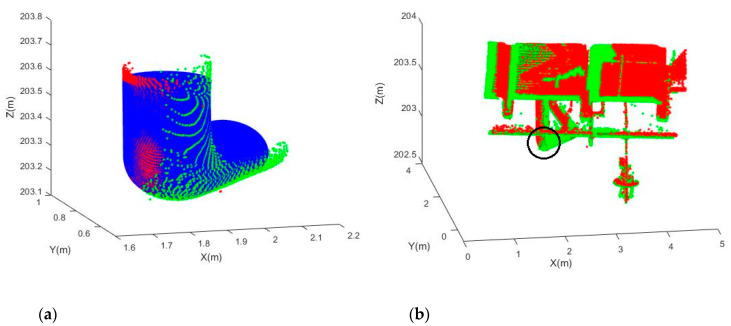
Registered point clouds: (**a**) The registered Joints G-H (red for Joint G, green for Joint H, and blue for the model); (**b**) The entire registered point cloud (the black circle shows the joint position).

Joints E and F are point clouds of the same joints obtained from two stations. After the fitting, their translational and rotational parameters were used for the registration based on Equation (11). Figure 15a shows that Joint F (green) is registered to the coordinate system of Joint E (red), and the simulated best fit joints (model) for Joint E are shown as well. It can be seen that the two point clouds match well with the model. Similarly, Figure 16a shows that Joint H (green) is registered to the coordinate system of Joint G (red). 

The resultant registered point clouds for Registration 1 (Joint E-F registration) and Registration 2 (Joint G-H registration) are shown in Figure 15b and Figure 16b, respectively. Their translational and rotational accuracies were computed based on independent check plane analysis [18] and are tabulated in Table 9. Registration 2 has better accuracy, reaching sub-decimeter and sub-degree levels for the translational and rotational parameters. This implies that the fitting of Joints G and H is more accurate than that of Joints E and F. In fact, Joints G and H are larger joints (the dimension is approximately double that of Joints E and F). From Table 8, it is found that Joint E has only about 40% of the number of observations of Joint F. Therefore, the fitting accuracy of Joint E is limited, and this degrades the registration accuracy. Overall, the results of the two registrations demonstrated that the model can be used to register point clouds with a fairly good accuracy depending on the error of the individual fitting to joints. 

#### 4.2.3. Mounting Bracket Detection

**Figure 17:**
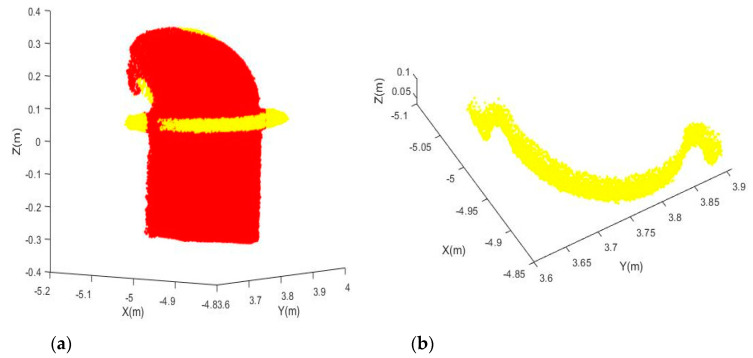
Detected mounting bracket from Joint B: (**a**) Point cloud (red) and points with higher residuals (yellow); (**b**) Extracted mounting bracket.

**Figure 18:**
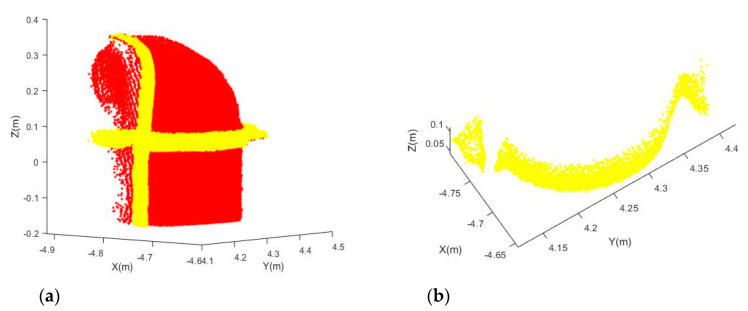
Detected mounting bracket from Joint C: (**a**) Point cloud (red) and points with higher residuals (yellow); (**b**) Extracted mounting bracket.

Figure 17a shows that the points with higher residuals are highlighted with yellow for Joint B. Those points are then grouped and filtered to extract the mounting bracket (Figure 17b). This example illustrated that the residuals obtained from fitting the observations to the model can be a powerful tool for us to detect any components that attach to the joints but do not belong to them. More residual-based applications can be potentially developed in the future. Similarly, Figure 18a shows that the points with higher residuals are highlighted with yellow for Joint C. It can be seen that there is a thin plastic tube (can be seen more clearly in Figure 9c) attached to the joint surface, and the mounting brackets have larger residuals. After performing the grouping and filtering processes, the mounting bracket (Figure 18b) was successfully detected.

## 5. Conclusions

In this paper, we proposed a novel geometric model and multiple model adaptions for several typical elbow joints commonly used in the piping systems for clean water supply and drainage. The model is in single equation format so that it equates the x, y, and z coordinates of joints obtained from laser scanner to a set of model parameters that describes the positions, orientations, and dimensions of joints in a 3D space, and the parameters can be readily estimated using the least-square technique. The model was verified by both simulated and real datasets, and two applications of the proposed model (point cloud registration and mounting bracket detection) were demonstrated and discussed. The results for the simulated datasets suggest that the model is robust to random noises for which the estimates only suffer from translational and rotational errors as low as 0.3 mm and 0.064°, respectively, even though ±0.02 m random errors were introduced to coordinates of joints with a 0.2-m diameter. The real dataset results are consistent with the simulation and suggest that the accuracy of the diameter estimate achieved 97.2%. The registration based on two point clouds of a single joint was proven workable, and the accuracy reached sub-decimeter and sub-degree levels for the translational and rotational parameters, respectively. It is known that the model can be used not only for monitoring the joints’ geometry but also for other applications such as point cloud registrations.

## Figures and Tables

**Table 1:** Details of the simulated datasets.

Joint	Type (Degree)	A/D Ratio	Flange
a	90°	1D	No
b	90°	1.5D	
c	90°	0.5D	
d	45°	1D	
e	45°	1.5D	
f	45°	0.5D	
g	90°	1D	Yes
h	45°	1D	

**Table 2:** Details of the real datasets.

Joint	Type (Degree)	Flange	Scanner	Application Demo
A	90°	Yes	Trimble SX 10	n/a
B	90°	No	Trimble SX 10	n/a
C	90°	No	Trimble SX 10	Mounting Bracket Detection
D	90°	Yes	Trimble SX 10	Mounting Bracket Detection
E	90°	Yes	Trimble SX 10	Point Cloud Registration
F	90°	Yes	Trimble SX 10	Point Cloud Registration
G	90°	No	Faro Focus^3D^	Point Cloud Registration
H	90°	No	Faro Focus^3D^	Point Cloud Registration
I	45°	No	Faro Focus^3D^	n/a
J	45°	No	Faro Focus^3D^	n/a

**Table 3:** RMSE of the translation parameters and the rotational parameters estimated from the fitting of the simulated datasets at noise levels from 0 mm to ±3 mm (D = 0.1 m).

Noise Level (mm)			0		±1		±2		±3	
**Joint**	**Type**	**A** **/D Ratio**	${\hat{\delta }}_{T}\left(mm\right)$	${\hat{\delta }}_{R}\left({}^{\circ}\right)$	${\hat{\delta }}_{T}\left(mm\right)$	${\hat{\delta }}_{R}\left({}^{\circ}\right)$	${\hat{\delta }}_{T}\left(mm\right)$	${\hat{\delta }}_{R}\left({}^{\circ}\right)$	${\hat{\delta }}_{T}\left(mm\right)$	${\hat{\delta }}_{R}\left({}^{\circ}\right)$
a	90°	1D	0	0	0	0.007	0	0.012	0	0.006
b	90°	1.5D	0	0	0	0.012	0	0.027	0.1	0.008
c	90°	0.5D	0	0	0.1	0.014	0	0.002	**2.4**	**4.007**
e	45°	1D	0	0	0.1	0.012	0.1	0.016	0.1	0.132
f	45°	1.5D	0	0	0	0.005	0.2	0.097	0.4	0.101
g	45°	0.5D	0	0	0.2	0.033	0.2	0.052	**1.9**	**3.090**
h	90°	1D	0	0	0	0.012	0.2	0.042	0.2	0.044
i	45°	1D	0	0	0.2	0.013	0.6	0.127	0.8	0.020

**Table 4:** RMSE of the translation parameters and the rotational parameters estimated from the fitting of the simulated datasets at noise levels from ±3 mm to ±6 mm (D = 0.2 m).

Noise Level (mm)			±3		±4		±5		±6	
**Joint**	**Type**	**A** **/D Ratio**	${\hat{\delta }}_{T}\left(mm\right)$	${\hat{\delta }}_{R}\left({}^{\circ}\right)$	${\hat{\delta }}_{T}\left(mm\right)$	${\hat{\delta }}_{R}\left({}^{\circ}\right)$	${\hat{\delta }}_{T}\left(mm\right)$	${\hat{\delta }}_{R}\left({}^{\circ}\right)$	${\hat{\delta }}_{T}\left(mm\right)$	${\hat{\delta }}_{R}\left({}^{\circ}\right)$
a	90°	1D	0.1	0.027	0.1	0.031	0.1	0.037	0.2	0.023
b	90°	1.5D	0	0.025	0	0.015	0.2	0.087	0.1	0.024
c	90°	0.5D	**0.3**	**0.045**	**1**	**0.181**	**n/a**	**n/a**	**n/a**	**n/a**
e	45°	1D	0.3	0.064	0.5	0.108	0.6	0.240	**1.9**	0.011
f	45°	1.5D	0.5	0.017	1	0.125	0.5	0.141	**1.1**	0.500
g	45°	0.5D	**1.7**	**1.772**	**3.7**	**3.082**	**n/a**	**n/a**	**n/a**	**n/a**
h	90°	1D	0.1	0.013	0.2	0.045	00.2	0.027	**0.4**	0.029
i	45°	1D	0.2	0.033	1.2	0.024	2	0.136	**2.5**	0.331

**Table 5:** RMSE of the dimensional parameters estimated from the fitting of the simulated datasets from 0 mm to ±6 mm (D = 0.2 m).

Noise Level (mm)			0	±3	±4	±5	±6
**Joint**	**Type**	**A** **/D Ratio**	${\hat{\delta }}_{AD}\left(m\right)$	${\hat{\delta }}_{AD}\left(m\right)$	${\hat{\delta }}_{AD}\left(m\right)$	${\hat{\delta }}_{AD}\left(m\right)$	${\hat{\delta }}_{AD}\left(m\right)$
a	90°	1D	0	0	0	0	0.1
b	90°	1.5D	0	0.1	0	0.1	0.2
c	90°	0.5D	0	0.2	0.3	n/a	n/a
e	45°	1D	0	0.4	0.1	0.5	0.3
f	45°	1.5D	0	0.1	0	0.1	0.2
g	45°	0.5D	0	0.7	1.6	n/a	n/a
h	90°	1D	0	0.2	0.3	0.3	0.4
i	45°	1D	0	0.8	0.8	0.9	1.3

**Table 6:** RMSE of the dimensional parameters estimated from the fitting of the simulated datasets from ±20 mm to ±80 mm (D = 0.2 m).

Noise Level (mm)			±20		±40		±60		±80	
**Joint**	**Type**	**A** **/D Ratio**	${\hat{\delta }}_{T}\left(mm\right)$	${\hat{\delta }}_{R}\left({}^{\circ}\right)$	${\hat{\delta }}_{T}\left(mm\right)$	${\hat{\delta }}_{R}\left({}^{\circ}\right)$	${\hat{\delta }}_{T}\left(mm\right)$	${\hat{\delta }}_{R}\left({}^{\circ}\right)$	${\hat{\delta }}_{T}\left(mm\right)$	${\hat{\delta }}_{R}\left({}^{\circ}\right)$
b	90°	1.5D	0.3	0.064	1.4	0.266	1.7	0.251	1.1	1.072
f	45°	1.5D	0.8	0.171	n/a	n/a	n/a	n/a	n/a	n/a

**Table 7:** Least-squares estimates and the precision of Joints (a) and (d) with different coverage values.

Joint	Type	A/D Ratio	Point Coverage	No. of Points	${\bar{X}}_{T}$ $\left(\mathrm{mm})\;{\bar{\sigma }}_{T}(\mathrm{mm}\right)$	${\bar{X}}_{R}\left({}^{\circ}\right)\;{\bar{\sigma }}_{R}\left({}^{\circ}\right)$	${\bar{X}}_{AD}$ $\left(\mathrm{mm})\;{\bar{\sigma }}_{AD}(\mathrm{mm}\right)$
			90° (25%)	11,790	682.500	45.697	75.000
					**9.54 × 10^−2^**	**6.29 × 10^−2^**	**1.14 × 10^−1^**
			180° (50%)	25,380	682.500	45.697	75.000
					**5.30 × 10^−2^**	**3.68 × 10^−2^**	**4.12 × 10^−2^**
a	90°	1D	270° (75%)	35,370	682.500	45.697	75.000
					**4.27 × 10^−2^**	**3.05 × 10^−2^**	**3.12 × 10^−2^**
			360° (100%)	47,160	682.500	45.697	75.000
					**3.67 × 10^−2^**	**2.62 × 10^−2^**	**2.62 × 10^−2^**
			90° (25%)	11,790	601.678	45.346	75.000
					**3.25 × 10^−1^**	**6.29 × 10^−2^**	**3.56 × 10^−1^**
			180° (50%)	25,380	601.678	45.346	75.000
d	45°	1D			**2.19 × 10^−1^**	**3.68 × 10^−2^**	**1.88 × 10^−1^**
			270° (75%)	35,370	601.678	45.346	75.000
					**1.78 × 10^−1^**	**3.05 × 10^−2^**	**1.51 × 10^−1^**
			360° (100%)	47,160	601.678	45.346	75.000
					**1.54 × 10^−1^**	**2.62 × 10^−2^**	**1.30 × 10^−1^**

**Table 8:** Mean least-squares estimates and the precision of Joints A to J.

Joint	Type	No. of Points	${\bar{X}}_{T}(\mathrm{mm})$ ${\bar{\sigma }}_{T}(\mathrm{mm})$	${\bar{X}}_{R}\left({}^{\circ}\right)$ ${\bar{\sigma }}_{R}\left({}^{\circ}\right)$	${\bar{X}}_{AD}(\mathrm{mm})$ ${\bar{\sigma }}_{AD}(\mathrm{mm})$
A	90°	18,966	5619.1	149.131	69.2
			**7.50 × 10^−2^**	**5.16 × 10^−2^**	**6.04 × 10^−2^**
B	90°	49,742	4391.4	103.613	129.1
			**4.79 × 10^−2^**	**7.62 × 10^−3^**	**4.34 × 10^−2^**
C	90°	29,155	1350.9	111.162	114.8
			**8.66 × 10^−2^**	**3.10 × 10^−2^**	**7.68 × 10^−2^**
D	90°	22,223	1638.4	15.757	61.0
			**5.97 × 10^−2^**	**2.53 × 10^−2^**	**5.31 × 10^−2^**
E	90°	8624	2583.376	42.562	49.2
			**1.45 × 10^−1^**	**6.46 × 10^−2^**	**1.28 × 10^−1^**
F	90°	23,085	1593.3	60.535	48.7
			**7.90 × 10^−2^**	**2.18 × 10^−2^**	**6.88 × 10^−2^**
G	90°	15,629	6811.9	65.881	89.9
			**5.54 × 10^−2^**	**7.27 × 10^−3^**	**5.65 × 10^−2^**
H	90°	13,569	6881.4	72.330	90.5
			**7.11 × 10^−2^**	**1.85 × 10^−2^**	**6.76 × 10^−2^**
I	45°	7387	3163.8	35.752	75.3
			**1.12 × 10^−1^**	**2.31 × 10^−2^**	**7.11 × 10^−1^**
J	45°	2356	6202.8	72.526	55.6
			**7.53 × 10^−1^**	**1.98 × 10^−1^**	**9.82 × 10^−1^**

**Table 9:** Translation and rotational errors for the registrations.

Registration	Joint	$E_{T}(\mathrm{mm})$	$E_{R}({}^{\circ})$
1	E-F	27	1.89
2	G-H	3.8	0.36
